# Prospective Phase II Multicenter Trial of Ablation after Breast Lumpectomy Added To Extend (ABLATE) Intraoperative Margins for the Sole Local Treatment of Breast Cancer

**DOI:** 10.1245/s10434-025-19008-8

**Published:** 2026-01-18

**Authors:** Kristalyn Gallagher, Sheldon Feldman, Julie Barone, Joshua Mammen, Robert Barone, Daniela Ochoa, Marilee McGinness, Thomas Frazer, Rebecca Viscusi, Julie E. Lang, Jeannette Lee, Laura L. Adkins, Sherry Johnson, Ronda Henry-Tillman, Soheila Korourian, V. Suzanne Klimberg

**Affiliations:** 1 Department of Surgery, University of North Carolina, Chapel Hill, NC USA; 2 Department of Surgery, Montefiore Medical Center, Bronx, NY USA; 3 Intermountain Health Saint Joseph Hospital, Santa Maria, CA USA; 4 Department of Surgery, University of Nebraska Medical Center, Omaha, NE USA; 5 Oncology Associates of San Diego at Sharp Memorial Hospital, San Diego, CA USA; 6 Department of Surgery, University of Arkansas for Medical Sciences, Little Rock, AR USA; 7 Department of Surgery, University of Kansas, Kansas City, KS USA; 8 Bryn Mawr Hospital, Bryn Mawr, PA USA; 9 Department of Surgery, Tucson Medical Center Health Cancer Center, Tucson, AZ USA; 10 Department of Surgery, Cleveland Clinic Lerner College of Medicine of Case Western Reserve University, Cleveland, OH USA; 11 Department of Biostatistics, College of Public Health, UAMS, Little Rock, AR USA; 12 Surgery, Anchorage, AK USA; 13 Department of Pathology, UAMS, Little Rock, AR USA; 14 Department of Surgery, Department of Breast Surgical Oncology, University of Texas Medical Branch, Galveston, and MDACC, Houston, TX USA

**Keywords:** Breast cancer, Lumpectomy, Conservative breast surgery, Radiation, Ablation, Radiofrequency, RFA

## Abstract

**Background:**

Excision followed by radiofrequency ablation (eRFA) is an intraoperative method that utilizes intracavitary hyperthermia to create an additional tumor-free zone around the lumpectomy cavity in patients with breast cancer, similar to partial breast irradiation. We hypothesized that intraoperative eRFA extends the “final” tumor-free margin, decreases local recurrence, and maintains cosmesis without the need for radiation (XRT).

**Patients and Methods:**

Patients with unifocal ER+PR+HER2− or DCIS tumors less than or equal to 3 cm with clinically negative nodes were included. After standard lumpectomy, the RFA probe was deployed 1 cm circumferentially into the walls of the lumpectomy cavity. RFA was performed at 100°C for 15 min, followed by validated intraoperative Doppler sonography. Pain and cosmesis were assessed with the Radiation Therapy Oncology Group (RTOG) scales.

**Results:**

A total of 242 subjects were accrued to the study, with a median follow-up of 44 months (range 12–96 months); 60% were invasive ductal cancer (IDC), and 33% were ductal carcinoma in situ (DCIS). The average size was 1.1 ± 0.6 cm (0.2–3 cm). Reexcision for positive margins was < 5%. In-breast recurrence rate was 2.9%. Breast pain at 6 months was 19% with RFA combined with XRT versus 1.7% with RFA alone (*p* < 0.05). Cosmesis was good or excellent in 89% of subjects.

**Conclusions:**

A majority of the subjects avoided whole breast XRT and mastectomy. Results indicate that eRFA, in lieu of XRT, is safe and effective, resulting in ~fivefold lower pain. By completing therapy in the operating room, eRFA can potentially enhance patient access and compliance, alleviate financial stress, and deliver superior cosmetic and quality-of-life outcomes.

The paradigm of lumpectomy followed by radiation (XRT) offers breast preservation while maintaining equivalent local and systemic recurrence compared with modified radical mastectomy.^1,2^ Some women cannot or do not want to undergo XRT. Women living far from radiotherapy facilities often choose mastectomy or undergo breast conservation surgery (BCS) but do not complete their recommended XRT course, potentially increasing the local recurrence rate (LRR).^3^

Approximately 75–90% of recurrent disease occurs within 1 cm of the primary tumor. Thus, irradiation of the peritumoral cavity (accelerated partial breast irradiation, APBI) has been developed and has provided equivalent local control rates to whole breast irradiation (WBXRT) in select patients.^4-8^ Furthermore, a large international, multicenter, prospective, randomized, noninferiority phase 3 trial (TARGIT-A trial) demonstrated that APBI via targeted intraoperative radiotherapy for BC was similar to WBXRT in terms of LRR and survival.^9,10^

Numerous studies have failed to identify a subgroup of patients with BC in whom radiotherapy can be completely avoided without a significant increase in local recurrence.^2,11,12^ Most notably, the NSABP B-21 randomized trial showed that in patients with favorable, hormone-sensitive BC ≤ 1 cm, the LRR was 16% with lumpectomy followed by tamoxifen alone, compared with 10% with lumpectomy + XRT, and 3% with lumpectomy + XRT + tamoxifen at 8-year follow-up.^2^ Additionally, multiple studies have demonstrated the superiority of negative margins in maintaining local control.^13^ Consequently, the rate of repeat surgeries necessary to achieve negative margins is unacceptably high (10–40%), prompting innovation for improvement in obtaining negative margins.^14-17^

Radiofrequency ablation (RFA) is approved by the U.S. Food and Drug Administration (FDA) for the ablation of subcutaneous tissue.^18^ Klimberg and colleagues, through a series of laboratory, preclinical, and clinical trials, established that excision followed by RFA (eRFA) of the lumpectomy cavity provides a consistent 1 cm zone of ablation around the cavitary tumor bed.^19-23^ Used in this way, eRFA was posited to reduce unnecessary reexcision as well as provide equivalent coverage of the pericavitary tumor bed, decreasing the LRR without XRT in patients with early stage BC. Building on these results, a long-term multi-institutional (seven sites) prospective nonrandomized phase II registry trial of excision followed by pericavitary RFA for local BCS was initiated.

## Patients and Methods

### Study Design

**Fig. 1:**
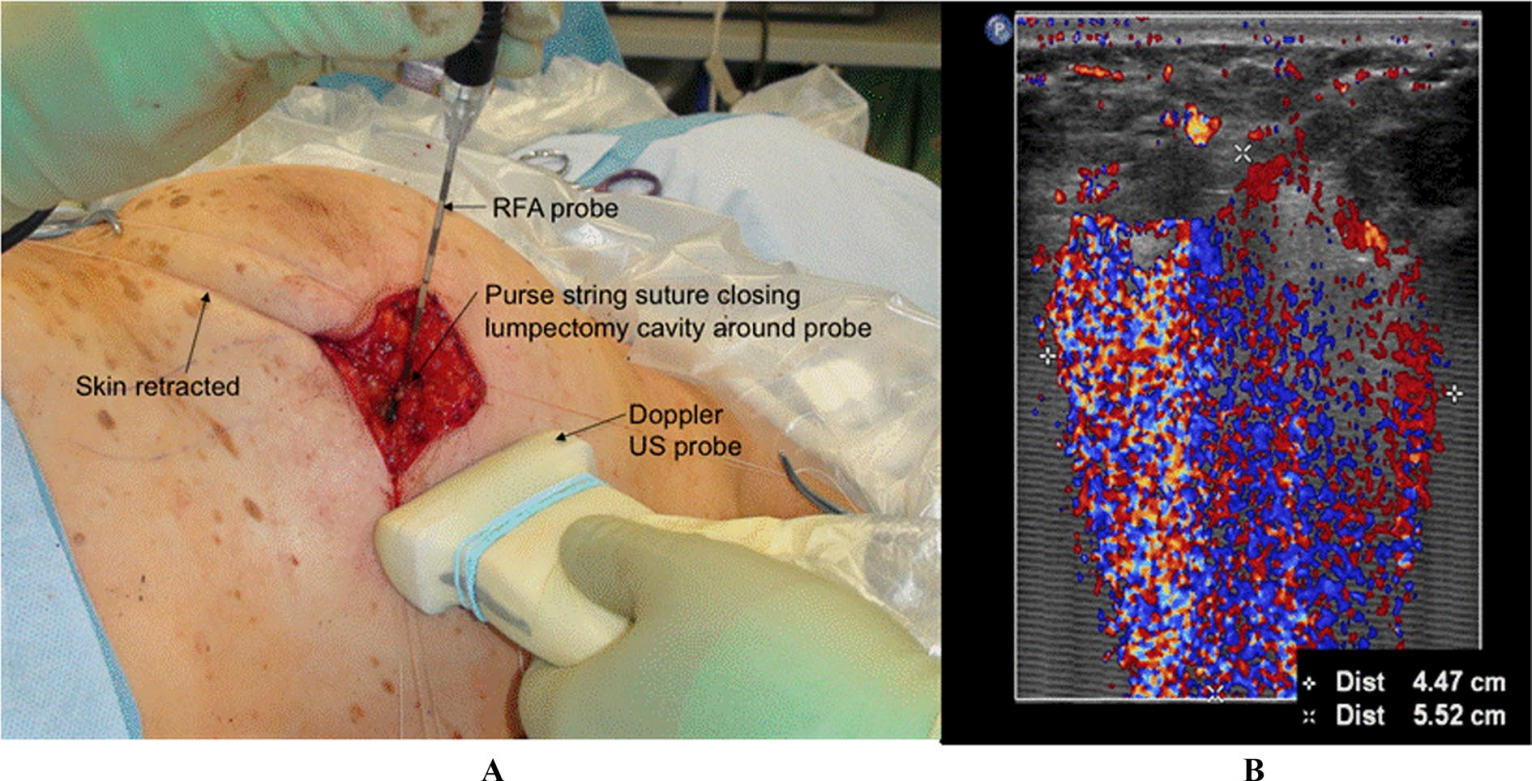
eRFA technique with intraoperative monitoring of the ablation zone; **A** intraoperative setup with a 12 MHz ultrasound probe used in Doppler mode for measuring ablation zone by visualizing movement of tissue caused by off-gassing of nitrogen; **B** Doppler signals that demonstrate an ablation zone of 4.47 cm (denoted by +) by 5.52 cm (denoted by *x*)

This trial was an Institutional Review Board (IRB)-approved, prospective, single-arm, multicenter study conducted at seven sites, with the total goal of accruing 250 postmenopausal subjects between 2010 and 2016. The protocol was registered with ClinicalTrials.gov and assigned NCT# 01153035 (Excision Followed by Radiofrequency Ablation for Breast Cancer, ABLATE). A Data Safety and Monitoring Board (DSMB) was in place. Patients > 50 years old with grade I/II invasive ductal cancer (IDC) cases that were unicentric, ER/PR+, and HER2−, or had a grade I/II ductal carcinoma in situ (DCIS) with any ER/PR status, ≤ 3 cm, > 1 cm from the skin, and clinically node negative, were accrued to the trial. Patients with prior cancer, bilateral cancer, invasive lobular cancer, grade III cancer, tumors > 3 cm or ER/PR− tumors were excluded from accrual. Patients with positive nodes received radiation. Patients received a lumpectomy followed by intracavitary RFA (Fig. 1A).^23^ The primary endpoint was LRR. Secondary endpoints included complications, reexcisions, scores on the acute and chronic Radiation Therapy Oncology Group (RTOG) scales, cosmesis, and quality of life (QOL) scales.

### Site and PI Training

Each site PI completed hands-on training at the University of Arkansas for Medical Sciences (UAMS) (by VSK, the overall Principal Investigator) and was anticipated to accrue 250 subjects. A central collection of Case Report Forms (CRFs) and histology reports was performed.

### Study Procedure

#### Preoperative Examination

Enrolled subjects were evaluated according to the standard of care, and a baseline Subjective Cosmetic Result Scale was completed. This subjective instrument determined how comfortable a subject was with their appearance (in general and breast-specific) prior to surgery.

#### Lumpectomy and Pathology

Standard lumpectomy was performed under general anesthesia using an image-guided^24^ or needle localization technique.^25^ After removal, the lumpectomy specimen was sent to pathology for routine processing. Shaved margins were allowed prior to the application of RFA. Reexcision was recommended after eRFA when final pathology demonstrated tumor at the inked margin of the resection specimen. In the case of a focally positive margin (defined as a single breach of one margin), the final decision regarding reexcision was made at the discretion of the treating site team.

#### Sentinel Lymph Node Biopsy

Sentinel lymph node biopsy (SLNB) was performed in subjects with invasive ductal carcinoma at the time of the initial procedure using technetium-99 and/or isosulfan blue dye for localization. Axillary reverse mapping (ARM) was used at some institutions.^26,27^ If the SLNB was positive for metastatic disease, an axillary lymph node dissection was performed.

#### Radiofrequency Ablation Procedure and Ultrasound Doppler Monitoring

Intraoperatively, following standard lumpectomy, subjects underwent intracavitary RFA (eRFA) using the RITA Medical Systems Starburst XL RFA probe (RITA Medical Systems, Mountain View, CA, USA).^19,21–23^ A purse-string suture was used to reduce the lumpectomy cavity to approximately 1 cm. The RFA probe was deployed into the cavity of the tumor bed to a depth of 1 cm, and the tissue surrounding the cavity was heated to 100 °C for 15 min with a power of 150 watts. A 12.5 MHz US probe in Doppler mode was used during surgery to track the ablation zone (Fig. 1B).^28^ After ablation, the wound was irrigated and closed.

#### Cosmetic Outcome and Quality of Life

Subjects were scheduled for follow-up 2 weeks after surgery, and every 6 months to 1 year for a period of 6 years. At each follow-up, administered scales included: the European Organization for Research and Treatment of Cancer (EORTC) Body Image Scale, the subjective cosmetic result scale (BII), and the RTOG acute and/or chronic scale.^29–31^ As a secondary outcome, patients also completed the Body Image Index or Subjective Assessment Scale.^32^ The EORTC Body Image Scale and the Body Image Index were to be administered at baseline, postoperatively at 2 weeks, and yearly.

The RTOG scoring criteria for acute radiation changes to the breast were completed by the physician, who recorded the difference in the skin of the treated breast before and after surgery.^29^ The late effects in normal tissues, subjective, objective, management, and analytic scales (LENT-SOMA)^33^ were administered between 6 months and 36 months. Each answer was scored from zero to four, depending on the severity of the symptoms, with a higher score indicating increasing severity. Retraction was measured as none, mild (10–25%), moderate (25–40%), severe (40–75%), or extreme (whole breast).

### Complications

Complications were defined as related to the instrument, such as failure of the device or inability to complete the ablation. Wound complications were defined by clinical presence of seroma, hematoma, burn, wound infection or abscess, or wound dehiscence. Serious adverse events (SAE) were defined by those occurrences that were life-threatening, resulting in hospitalization and/or death.

### Statistical Methods

Data were collected and analyzed in a prospective database using Microsoft Excel (Microsoft Corporation, Redmond, WA). Descriptive statistics were performed on age, tumor size, margins, and recurrences using Fisher’s exact probability. Disease-free survival was defined as the time from surgery to either BC recurrence or death. Computations were carried out using Stata for Windows, Release 10 (StataCorp LP; College Station, TX).^34^

To determine whether body image deteriorated after RFA, the modified versions of the pretreatment and 6-month posttreatment scales were compared using the Wilcoxon signed-rank test. Mann–Whitney tests and Spearman correlations were used to determine whether the posttreatment EORTC Body Image scores were associated with clinical or demographic characteristics.

## Results

### Study Population

Data were collected from 3 September 2010 to 1 November 2022, from seven institutions. A total of 267 women were screened for the study at seven different sites; 25 subjects were screen failures, leaving a study population of 242 subjects. All site teams received training at UAMS in Little Rock, AR. Accrual of subjects by institution ranged from 8–89 subjects.

The median follow-up time was 44 months (range 12–96 months). The average age of the total group of participants was 67.0 ± 8.6 years, with the majority (89%) being Caucasian, followed by Black (5%), Hispanic (2%), and Asian (2%) participants (Table1).

**Table 1:** Demographic characteristics and medical history

	*N* = 242
Age in years, mean (SD)	67.0 (8.6)
Body mass index, mean (SD)	28.0 (6.0)
*Race/ethnicity, n (%)*	
White	216 (89)
Black	11 (5)
Hispanic	5 (2)
Asian	4 (2)
Other	3 (1)
Unknown	3 (1)
*Medical history, n (%)*	
Smoking	78 (32)
Alcohol	119 (49)
Cardiovascular disease	125 (52)
Pulmonary disease	28 (12)
Diabetes	14 (6)
Hepatic disease	6 (2)
Renal failure	3 (1)
Collagen vascular disease	0
Dermatological disease	23 (10)
Hematological disease	6 (2)
Allergies/sensitivities	116 (48)
Infectious disease	2 (1)
Psychiatric disease	33 (14)

### Disease Characteristics

Most participants (60%) had IDC (Table 2), one-third had DCIS, and 98.3% of all were ER/PR+. A total of 16 individuals (7%) had missing data as to the type of cancer. The average tumor size was 1.1 ± 0.6 cm (0.2–3 cm). Reexcision for positive margins occurred in five subjects (< 5%). Breast/axillary infection occurred in 3 subjects. There were nine mastectomies due to positive margins, extensive disease (greater than 5 cm), and recurrence, and one due to severe radiation reaction.

**Table 2:** Disease characteristics

	*N* = 242(%)
*Laterality, n (%)*	
Left breast	121 (50)
Right breast	120 (50)
Missing	1 (< 1)
*Histology, n (%)*	
Invasive ductal	146 (60)
DCIS	80 (33)
Missing	16 (7)
ER/PR+	238 (98.3)
Her2neu+	9(3.7%)
Largest diameter of tumor (cm), mean (SD)	1.08 (0.6) *N* = 222
*Tumor location, n (%)*	
Upper outer quadrant	92 (38)
Lower inner quadrant	15 (6)
Lower central	8 (3)
Lower outer quadrant	17 (7)
Central	5 (2)
Inner central	11 (5)
Upper inner quadrant	40 (17)
Upper central	32 (13)
Outer central	21 (9)
Missing	1 (< 1)

## Radiation Therapy

In this risk-adjusted model, XRT was added when SLNB was positive; 20% of the cohort received XRT. Retraction of the breast greater than 25% was reported in 6% of cases and was only noted in those who received XRT. Breast pain at 6 months was 19% with eRFA + XRT versus 1.7% with eRFA alone (*p* < 0.05). Cosmesis was good or excellent in 89% of patients. QOL did not change after eRFA.

### Systemic treatment

Of the total subjects, 147 (60.7%) received endocrine therapy, and 23 (9.5%) received chemotherapy. The remaining subjects did not receive systemic therapy.

### Complications

Two participants experienced device failures, and their ablations were prematurely stopped. Complications included five seromas/hematomas, two skin edge burns, six wound infections (2.4%), and two wound dehiscences, as well as two axillary seromas and one pneumothorax (not requiring intervention and not felt related to RFA), for a total complication rate of 7.4%.

### Recurrences

The median follow-up for the group was 44 months (range 19.8 months). LRR was 2.9% for the entire group, 3.1% for eRFA alone, and 1.7% for those who received additional XRT (*p* = ns).

Of the seven recurrences, four were from subjects with DCIS and three were from subjects with IDC. Two of these were screening failures but reported here as intent-to-treat. Of all recurrences, 4/7 took the recommended hormonal therapy.

One DCIS recurred at 12 months as a DCIS on an aromatase inhibitor but was felt to be a second primary occurring > 5 cm from the primary site and was treated with lumpectomy. Another DCIS on an aromatase inhibitor recurred as a triple negative grade 3 IDC at 18 months directly in front of the eRFA cavity and was felt to be a missed primary. The third DCIS was grade 3 ER−PR+Her2neu+ that occurred at 24 months as a DCIS at the lumpectomy site just beyond the eRFA ablation. It was a screening failure as well. The fourth DCIS, which was treated with radiation, recurred as a DCIS at the lumpectomy site at 54 months and had been treated with radiation but not hormonal therapy.

IDC recurrence at 6 months was a patient who on final pathology had a grade 3 ER negative and PR weakly positive breast cancer recurred with distant disease without LRR by the 6-month follow-up, and refused any treatment and was considered a screening failure. The patient died at 12 months. At 24 months, a patient with grade 2 ER+PR+Her2neu− IDC recurred with contralateral axillary lymph nodes. The third IDC recurred at 50 months and was an invasive mucinous ER+PR+Her2neu− IDC that occurred 3 cm away from the RFA site as a DCIS. The patient did not take the recommended hormonal therapy.

### Acute Breast Toxicity

On the basis of the RTOG acute scale, breast assessments for most participants were excellent (Table 3).

**Table 3:** RTOG acute scale (obtained at 2 weeks, *N* = 234)

Breast assessment	*N* (%)
Excellent	122 (52)
Good	87 (37)
Fair	24 (10)
Poor	1 (< 1)
*Breast skin assessment*	
No change from before surgery	157 (67)
Faint redness or inflammation, dry peeling of skin, decreased sweating	57 (24)
Tenderness, bright redness and inflammation, patchy and moist peeling of skin, moderate swelling	18 (8)
Severe moist peeling of skin, severe swelling	1 (< 1)
Open wound, bleeding, or unpleasant odor	1 (< 1)

### Chronic Breast Toxicity

Subjects scored their quality of breast surgery outcome in 6-month intervals using the LENT-SOMA evaluation. Pain and edema were infrequently reported in subjects receiving RFA alone compared with those receiving XRT. There was significant extended pain noted when radiation was added to breast surgery. At 6 months, in those subjects treated with eRFA alone without XRT, 33/172 (19.2%) had grade 1 pain and 3/172 (1.7%) had grade 2 pain. Of those treated with XRT, 20/56 (36%) had grade 1 pain, and 8/56 (14,3%) had grade 2 pain.

At 1 year, 32/213 (15%) of evaluable patients had grade 1 fibrosis, 56/213 (26%) had grade 2, and 1/213 had grade 3 (0.5%). At 2 years, 18/180 (10%) had grade 1 fibrosis and 54/180 (30%) had grade 2 fibrosis. At 3 years, the prevalence of grade 1 fibrosis was 15/136 (11%), and that of grade 2 was 29/136 (21%). At 4 years, the prevalence of grade 1 fibrosis was 8/89 (9%), and that of grade 2 was 16/89 (18%). At 5 years, the rates were 2/25 (8%) and 4/25 (15%) for grades 1 and 2 fibrosis, respectively. Fibrosis in the eRFA group was reported at the site for eRFA patients and diffusely for those receiving whole breast XRT, but did not differ between the groups otherwise (*p* = ns).

Telangiectasia and lymphedema occurred infrequently and only in those subjects treated with radiation. Retraction of 10–15% of the breast was reported in 11% of eRFA and 19% of BCS+XRT participants. Using the LENT-SOMA scale,^33^ retraction of greater than 25% was reported in 6% of subjects, and only in those receiving XRT.

### Cosmetic Outcome

#### Evaluation at 2 Weeks

A total of 234 subjects scored their cosmesis at their postoperative visit after eRFA and prior to any other therapy. Using the RTOG acute radiation morbidity scoring criteria, 122 subjects (52%) rated their breast appearance as excellent, 87 (37%) as good, and 24 (10%) as fair. One subject scored her breast as poor after an infectious complication. Of the 234 patients, 89% scored their breasts as excellent or good. (Table 3)

#### Quality of Life (Body Image)

Of the 134 patients who provided evaluable body image questionnaires at baseline, 112 subjects (83.5%) provided evaluable data at the 6-month follow-up. Those who did not complete the follow-up assessments were more likely to have received radiotherapy (*p* = 0.02). They did not differ from protocol completers on other sample characteristics (e.g., tumor stage, grade, age, ethnicity, number of comorbidities).

There was no significant change in scores from pretreatment (median = 1) to 6-month follow-up (median = 1) on the modified EORTC Body Image Scale. Similarly, there were no significant changes in the modified Body Image Index from pretreatment (median = 20) to 2-week follow-up (median = 20), or from pretreatment to 6-month follow-up (median = 21).

At the 6–12-month follow-up, subjects in the current study (median = 1) scored significantly better on the EORTC Body Image Scale relative to BC patient norms (median = 6.00; one-sample Wilcoxon signed-rank test, *p* < 0.00001). When these comparisons were limited to norms from subjects treated with wide local excision (i.e., the most favorable group, median = 3.00), eRFA patients in the current sample still scored significantly better (one-sample Wilcoxon signed-rank test,* p* = 0.003).

Younger age, but not other clinical or demographic variables, was significantly correlated with worse scores on the EORTC Body Image Scale (Spearman’s *r* = −0.25).

The LENT-SOMA scale, commonly used by the RTOG to score both subjectively and objectively symptoms of long-term cosmesis and breast size post-radiation (*n* = 17), yielded a score of 5.4 ± 3.6 versus eRFA (*n* = 68), 1.5 ± 1.8, *p* = 0.0001.

#### Imaging

**Fig. 2:**
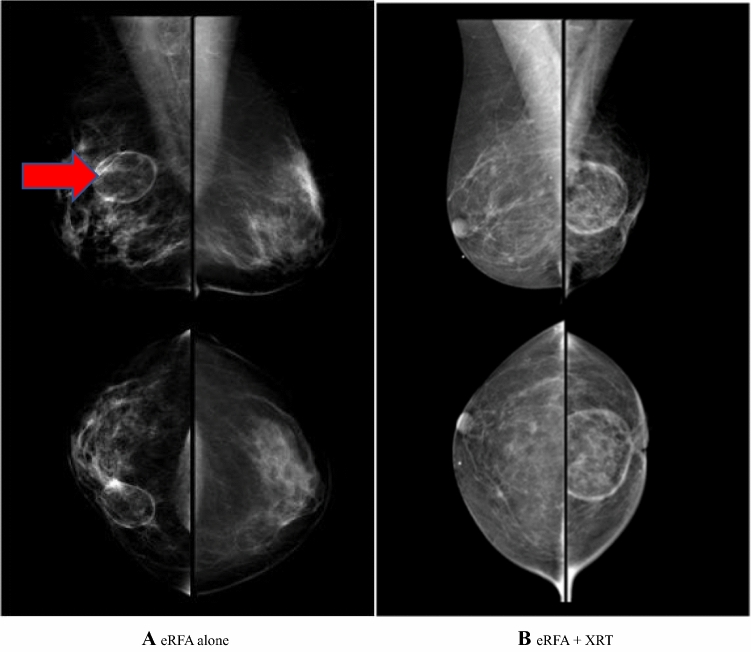
Mammogram after eRFA with and without XRT; representative comparison of mammographic changes 2 years posttreatment; **A** ablation zone (arrow) on right breast; equivalent size of the breasts on the mirror image views of lateral oblique (top) and cranial caudal views (bottom) can be seen; **B** mirror images of right and left breast showing significant shrinkage of left breast after radiation on both the lateral oblique (top) and cranial caudal (bottom) views

After eRFA, a ring of fat necrosis was visible on mammogram in 93% of cases (Fig. 2A). Significantly after XRT, but very minimally with eRFA, there was a reduction in breast size, resulting in a notable size discrepancy (Fig. 2B). Three subjects required later core needle biopsies for BIRAD 4 mammogram findings. All of these biopsies were benign (*p* = ns).

At 18 months, 9/24 subjects (38%) with XRT had breast shrinkage (ranging from 10% to 40%) versus 6/51 (12%) subjects with eRFA alone (ranging from 10% to 25%) (*p* = 0.01).

### Serious Adverse Events, DFS, and Overall Survival

#### Serious Adverse Events

A total of 16 serious adverse events (SAEs) and 5 deaths were reported (Table 4). Four of the SAEs were attributed to eRFA. One was a burn, and the others were infection or poor wound healing. The all-cause mortality rate was 2%, with a median follow-up of 44 months. Disease-free survival was 97.2%.

**Table 4:** Serious adverse events

Time	Event	Outcome
Week 2	Hematoma/infection/pathology	Mastectomy/hospitalization
Week 2	Pathology	Mastectomy/hospitalization
Week 2	Wound dehiscence	Wound revision
Week 2	Seroma/infection	Hospitalization
Week 2	Cellulitis	Hospitalization
Week 2	Cellulitis	Hospitalization
Month 2	Mastectomy	Hospitalization
Month 2	Mastectomy/recurrence	Hospitalization
Month 2	Mastectomy/recurrence	Hospitalization
Month 2	Burn/cellulitis/abscess	Hospitalization
Month 2	Mastectomy/positive margins	Hospitalization
Month 2	Mastectomy/positive margins	Hospitalization
Month 6	Mastectomy/unknown	Hospitalization
Month 6	Mastectomy for multicentricity	Hospitalization
Month 6	Cellulitis	Hospitalization
Month 18	Mastectomy	Unknown
Month 12	Metastatic disease	Death
Month 24	Stage 4 colon cancer	Death
Month 28	Metastatic disease	Death
Month 31	Cardiac event	Death
Month 36	COPD	Death

## Discussion

For many women diagnosed with early stage BC, breast-conserving surgery (BCS) is the treatment of choice. However, the current standard of care often involves follow-up radiation and systemic therapy that can be beyond the means of the patient, emotionally and/or physically. The ABLATE study explores a promising alternative: intraoperative radiofrequency ablation (eRFA), a technique designed to address the significant challenges associated with traditional BC treatment, including the burden of radiation therapy, and the associated financial and cosmetic costs. By demonstrating that eRFA can achieve similar local control with less morbidity and improved patient satisfaction, this research presents eRFA as a potential alternative treatment paradigm.

Beyond the surgical risks, traditional adjuvant radiation therapy (XRT), a standard component of BCS, presents its own set of hurdles. While randomized trials have proven that XRT is as effective as a total mastectomy in terms of long-term survival, it carries several notable drawbacks. A significant number of patients, as many as 30%, do not complete their prescribed radiation regimen, often due to the inconvenience and cost of daily treatments over several weeks.^35^ For many, particularly those living in rural areas with limited access to a radiation center, a mastectomy may seem like a more practical choice.^3^ Furthermore, XRT can negatively impact a patient’s quality of life and cosmetic outcome, leading to long-term issues such as a small increased risk of heart disease, breast retraction, and chronic pain.^36^ The ABLATE multicenter trial investigated the ability of eRFA to deliver definitive local therapy at the time of initial surgery, thereby eliminating the need for subsequent treatments and enhancing patient compliance. This is a particularly valuable benefit, especially for individuals in rural and underserved communities. As a one-time procedure performed solely by the surgeon, eRFA can significantly lower both patient and healthcare system costs by avoiding the need for multiple providers to provide local treatment, surgeries, and prolonged daily treatments resulting in financial toxicity. This multicenter trial compares favorably with the single institution trial results, demonstrating that the procedure is easily adopted and performed, resulting in low recurrence rates of 3% or less.^19–23^ Additionally, even if recurrence were to occur, eRFA does not preclude subsequent treatment options, such as additional surgery or traditional radiation, as the breast tissue has not been broadly radiated.

eRFA compares favorably with the findings of other partial breast trials, such as TARGIT, which has shown that intraoperative radiation is a viable alternative to whole-breast XRT. The data suggest that delivering definitive therapy immediately after tumor removal is a superior approach, as the precise treatment area is most easily identified at that time.^37–39^ Additionally, coordination of care is eased, as eRFA requires only the expertise of the surgeon.

The study’s findings on clinical outcomes are encouraging. With a median follow-up of 44 months, the overall survival rate was 98% and the disease-free survival rate was 97.2%. LRR for the entire group was 2.9%, a figure that compares favorably with data from our previous single-institution trials and other contemporary trials.^19–23^

In terms of toxicity and cosmetic outcomes, the eRFA group consistently demonstrated superior results compared with those who received XRT. At 18 months, breast retraction was significantly greater in the XRT group, with some patients experiencing a 10–40% reduction in breast size. By contrast, the eRFA-alone group had minimal size reduction. Chronic pain and fibrosis were also substantially less frequent in the eRFA group. For example, at 6 months, grade 2 pain was reported by 14.3% of subjects who received XRT, but only 1.7% of those who received eRFA alone.

Patient-reported outcomes further highlight the benefits of eRFA. At the 2-week evaluation, a combined 89% of subjects rated their breast appearance as “excellent” or “good” on the RTOG acute cosmesis scale, a figure that compares favorably with the 70% reported in the literature for irradiated breasts.^40^ These positive outcomes also translated into a better quality of life. The study found no significant increase in body image problems after eRFA, with patients scoring significantly better on body image scales compared with norms for patients with BC.

Beyond the clinical and cosmetic advantages, eRFA presents a compelling economic argument. The estimated cost for a full course of traditional XRT can exceed $47,000. The eRFA procedure, by contrast, is estimated to cost around $6295. This substantial cost reduction could lead to a significant decrease in the overall financial burden of BC treatment. For patients who live in rural areas or have limited financial resources, avoiding prolonged radiation and potential mastectomy can make a life-altering difference. By providing a safe, effective, and affordable one-time treatment option, eRFA has the potential to increase treatment compliance and make breast-conserving surgery a more accessible option for a broader patient population.

### Limitations and Future Directions

While the findings are promising, the study acknowledges its limitations. As a phase II, nonrandomized phase II registry trial, the data provide valuable insights but do not constitute definitive proof of equivalency to traditional radiation. The ABLATE study does not directly compare eRFA with XRT alone or with IORT alone. The number of missing patient records was a limitation of the registry design. However, the study’s size is notable, being as large or larger than several of the original brachytherapy and intraoperative radiation trials that target the same favorable patient population, leading to a consensus on APBI.^41^ The data gathered from this work will be crucial for powering a future, larger-scale, prospective, and randomized phase III trial to definitively determine whether eRFA is as effective as standard radiation therapy in preventing local recurrence.

## Conclusions

This research suggests that the BCS + eRFA approach holds great promise as a safe, low-cost, and low-morbidity alternative for a carefully selected group of patients. It has the potential to change the BC treatment paradigm, leading to the deescalation of radiation for those who are unlikely to benefit from it. By completing therapy in the operating room solely by the surgeon, eRFA can reduce provider care coordination, enhance patient access and compliance, alleviate financial stress, and potentially deliver equivalent or better cosmetic and quality-of-life outcomes.
